# Evaluation of BDNF as a Biomarker for Impulsivity in a Psychiatric Population

**DOI:** 10.3390/diagnostics10060419

**Published:** 2020-06-20

**Authors:** Stanislav Pasyk, Nitika Sanger, Flavio Kapczinski, Zainab Samaan

**Affiliations:** 1 Department of Psychiatry and Behavioural Neurosciences, McMaster University, Hamilton, ON L8S 4L8, Canada; stanislav.pasyk@medportal.ca (S.P.); kapczinf@mcmaster.ca (F.K.); 2 Medical Sciences Graduate Program, McMaster University, Hamilton, ON L8S 4L8, Canada; sangern@mcmaster.ca; 3 St. Joseph’s Healthcare Hamilton, Hamilton, ON L8N 4A6, Canada; 4 Department of Health Research Methods, Evidence, and Impact, McMaster University, Hamilton, ON L8S 4L8, Canada; 5 Population Genomics Program, Chanchlani Research Centre, McMaster University, Hamilton, ON L8S 4L8, Canada

**Keywords:** BDNF, risk assessment, impulsivity

## Abstract

Impulsivity is an important risk factor for suicide and therefore, identifying biomarkers associated with impulsivity could be important in evaluating psychiatric patients. Currently, assessment of impulsivity is based solely on clinical evaluation. In this study, brain-derived neurotrophic factor (BDNF), a nerve growth factor, was evaluated as a potential biomarker for impulsivity. We hypothesize that elevated BDNF may result in aberrantly high neurobiological activation, promoting impulsive behaviours. A total of 343 participants were recruited for the study and were divided into two groups, (i) elevated suicide risk (participants admitted to hospital with a recent suicide attempt), and (ii) average suicide risk (non-psychiatric participants and psychiatric participants without a history of suicide attempts). Impulsivity was measured by the Barratt Impulsiveness Scale, and serum BDNF levels were obtained. A regression analysis was performed to identify associations between BDNF and impulsivity. We identified a subtle but significant positive association between BDNF and impulsivity in the average risk for suicide group (B = 0.189, *p* = 0.014). The same association was not reproduced in the elevated risk group B = −0.086, *p* = 0.361). These findings lay the foundation to further explore the utility of BDNF as a biomarker for impulsivity to allow for early intervention.

## 1. Introduction

Impulsivity is a behavioural attribute defined by the tendency of an individual to act with little forethought or consideration of consequences. Impulsivity is an important behavioural facet for mental health as it plays a fundamental role in several different psychopathologies including but not limited to: attention deficit hyperactivity disorder (ADHD), substance use disorder, bipolar disorder and borderline personality disorder [1,2,3]. Impulsiveness leads to a higher likelihood of executing behaviours that individuals may later regret, including excess spending, gambling, risky sexual behaviours and dangerous driving. The sequelae of such behaviours can contribute to major life stressors, predisposing an individual to painful consequences and premature loss of life. Perhaps the most significant contribution impulsivity has in mental health is as a significant risk factor for suicide [4]. As such, it would be useful to develop objective means to identify individuals with high impulsiveness, and consider increased monitoring these at-risk individuals as part of an overall prevention strategy in the domain of mental health. 

Behavioural attributes such as impulsivity are underpinned by biological, psychological, social and environmental factors. The neurobiological basis for impulsivity is not comprehensively known, however the prefrontal cortex region (PFC) and its connected areas including the basal ganglia are the most heavily implicated in major studies of impulsivity [5]. With a biological underpinning, it is reasonable to hypothesize that a biomarker can be identified which is associated with impulsivity. To date, no such biomarker has been consistently identified for impulsivity. Clinicians who factor patient impulsivity into risk assessments are forced to rely solely on clinical judgment based on a subjective history and past behaviours. One clinical tool for the evaluation of impulsivity is a self-reported scale of impulsive behaviours called the Barratt Impulsivity Scale (BIS) [6,7]. Despite the wide use of this scale, as with any self-reported scale, there are limitations to the accuracy and utility. There is some evidence that the BIS score may be a predictor of suicide [8]. In contrast, one previous study found that BIS alone is unable to differentiate individuals with suicide ideation only from those with suicide attempts [9], highlighting the need for a more objective measure to identify trait impulsivity.

Brain-derived neurotrophic factor (BDNF) is a member of the neurotrophin family of proteins that plays many essential roles in the neuron, including neurogenesis, nerve growth, neuroplasticity and neurotransmission [10]. BDNF levels are measurable in serum samples, though these levels are affected by many factors including sex, age, body mass index (BMI), physical activity, nutrition, menstrual cycles and stress [10]. Despite multiple variables influencing serum BDNF, it has gained interest as a biomarker in mental health, and have been implicated in various psychopathologies such as depression, schizophrenia and Alzheimer’s disease [11,12,13]. Low levels of BDNF have been hypothesized to contribute to decreased neurobiological activity associated with depression [14]. BDNF is thought to play a role in intracellular signaling in brain regions associated with depression, including the hippocampus, prefrontal cortex, nucleus accumbens and amygdala [15]. BDNF depletion studies in mice have linked low BDNF to dysfunction in the circuitry of the hippocampus and amygdala [16,17]. Knock-out of Tropomyosin receptor kinase B (TrkB), a BDNF receptor, leads to increased risk-taking behaviour in mice, suggesting that this signaling pathway may play an important role in impulsivity [18]. We hypothesize that high levels of BDNF may represent an increase in neurobiological activity which might contribute to impulsive behaviour by preventing higher level processing of decision making that might hinder the forethought and consideration of consequences. If this hypothesis is correct, BDNF would make an important candidate for investigation as a biomarker for impulsivity.

Our objectives are:

1. Investigate the association between BDNF serum level and BIS impulsivity score in the total sample.
2. Perform a subgroup analysis to explore the association between BDNF and BIS score in high versus average risk of suicide groups.

## 2. Materials and Methods

### 2.1. Setting and Participants

Eligible participants for this study were recruited between March 2011 and November 2014 in Hamilton, Ontario for the Study of Determinants of Suicide Conventional and Emergent Risk (DISCOVER), a case-control study designed to investigate risk factors of attempted suicide [8,19]. Participants were deemed eligible to be included in the study if they met the following criteria: aged 18 years or older, able to provide written consent, able to communicate in English. The case group was comprised of individuals admitted to hospital with a serious suicide attempt within 1 month of admission to hospital. A serious suicide attempt was defined as one requiring admission to hospital for medical and/or psychiatric intervention. The control group was comprised of a variety of participants with no current or past history of suicide attempts. This group included participants: (i) admitted to psychiatric wards for reasons unrelated to suicidality; (ii) attending the general hospital for minor medical procedures or admitted for medical/surgical causes; (iii) attending outpatient clinics; or (iv) community members not seeking medical care [19].

Exclusion criteria included inability to provide informed consent, or an inability to follow study procedures. Candidates for the study were approached by trained research personnel and provided with detailed information about the study. Candidates agreeing to participate were asked to provide written informed consent. Participants underwent a structured interview, during which the 30-item Barratt Impulsiveness Scale [7] was applied and blood samples were collected after an overnight fast.

Community participants were recruited via distributed advertisements in hospitals, university and community settings. Candidates interested in participating who were able to provide informed consent were subjected to screening and a diagnostic interview. Where available, the medical charts were assessed for confirmation of the patient provided medical history.

For the purpose of this study we grouped the study participants based on their suicide risk into 2 groups: (i) elevated suicide risk (participants admitted to hospital with a recent suicide attempt), and (ii) average suicide risk (non-psychiatric participants and psychiatric participants without a history of suicide attempts). This study was approved by the Hamilton Integrated Research Ethics Board at St. Joseph’s Healthcare sites (#11-3479, 21 December 2016) and Hamilton Health Sciences sites (#10-661, 21 December 2016).

### 2.2. Data Collection and Instruments

Study questionnaires were designed for the acquisition of participant information in a face-to-face interview format. The questionnaires were administered by trained research personnel in both hospital and community settings. The data were entered into case report forms designed for the study.

### 2.3. Statistical Analysis

STATA version 13 was used to perform statistical analyses. We assessed the association between impulsivity score and BDNF serum level in the whole sample using multivariable linear regression analysis with impulsivity score as the dependent variable and BDNF serum level as the independent variable. A normal distribution was observed in histograms for BIS score, thus we conducted our analyses using parametric tests. We included age, sex and the risk group category into the model as explanatory variables. We then performed a subgroup analysis to investigate the association between BDNF and impulsivity based on the suicide risk category.

### 2.4. Laboratory Methods

Samples were collected and handled as described in Samaan et al. [19]. Two-hour fasting blood samples were collected, allowed 30 min clotting time, and spun at 1500 g for 15 min ensuring adequate blood separation. Samples were aliquoted in cryovials within 2 h of collection and stored at −196 °C (liquid nitrogen) at the Clinical Research and Clinical Trial Laboratory in Hamilton, Ontario. Serum BDNF levels were measured in the samples with a Quantikine^®^ ELISA Human BDNF Immunoassay (R&D Systems Inc., Minneapolis, MN, USA). Sample analysis was conducted in a blinded fashion to avoid bias.

This study is reported in accordance with the *Strengthening the Reporting of Observational Studies in Epidemiology* (STROBE) guidelines [20].

## 3. Results

### Study Sample Characteristics

**Figure 1:**
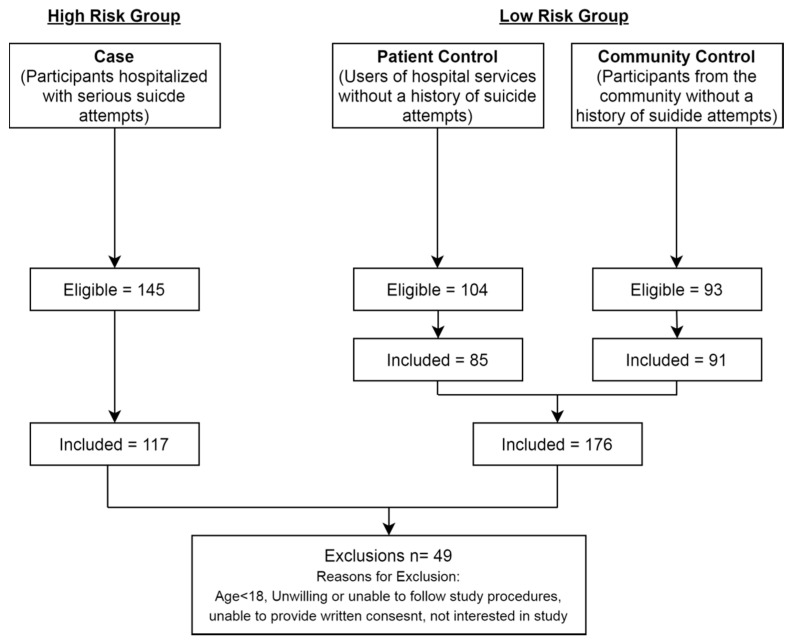
Flow diagram outlining study participants, adapted and repurposed from original study recruitment plan from Samaan et al. with permission [19].

The study sample comprised 293 participants including 176 at low risk and 117 at high risk for suicide. Figure 1 represents the participant recruitment flowsheet, highlighting the number of participants included in the final sample. The overall characteristics of the participants in the study has been characterized in Table 1. The mean age of the participants was 45.69 (SD = 15.52) with 48.8% of participants being female. The mean BMI of participants was 34.42 (SD = 17.38). The mean score on the BIS scale was 65.72 (SD = 8.00).

A comparison of the low risk and high risk sample characteristics is shown in Table 2. The age distribution between the two groups was comparable, with a mean age in the high risk group was 44.96 years (SD = 14.88) and for the low risk group 46.17 years (SD = 15.96). Each sample had a generally similar proportion of females and males, although the high risk group did have more males (45.30% female for high risk and 50.13% female for low risk). The mean body mass index of the two groups was not statistically different, with a mean BMI of the high risk group of 32.92 (SD = 16.44) as compared the 34.62 (SD = 17.57) for the low risk group. Despite the inherent differences in suicidality between the two groups, the serum BDNF was not significantly different (*p* = 0.947), nor were the BIS scores (*p* = 0.731). Demographic data including employment status, marital status and number of children are also highlighted in Table 2. There were generally few significant differences between the high and low risk groups, though the employment status between groups was significantly different. As expected, given the nature of the high and low risk groups, the frequency of psychiatric diagnoses and psychopharmacological treatment was ubiquitously significantly different. The ethnicity of participants varied between groups, however each group has a vast majority of participants of European ethnicity.

Table 3 summarizes how the variables of age, sex, BMI and BDNF correlate with the dependent variable of BIS score in the pooled sample population. Sex, BMI and BDNF were not significantly associated with BIS score. We identified a significant negative correlation between age and impulsivity in our combined population (beta = −0.168, *p* = 0.004), suggesting a tendency towards a decline in self-reported impulsivity as one ages.

**Figure 2:**
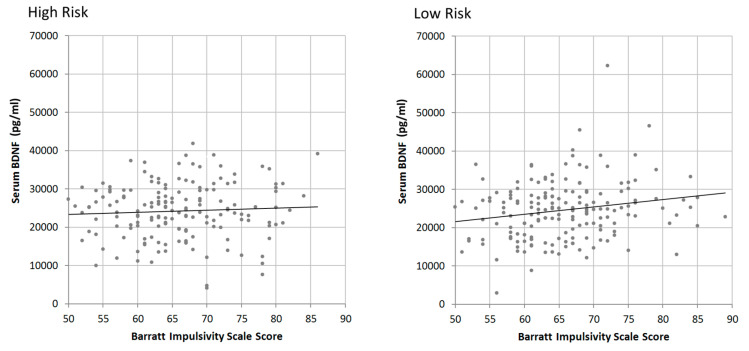
Scatter plot comparing serum BDNF (pg/μL) and BIS score in high risk (**left**, *n* = 117, beta = −0.086, *p* = 0.361) and low risk (**right**, *n* = 176, beta = 0.189, *p* = 0.014) populations.

Table 4 and Table 5 examine each variable’s association with BIS scores in the low and high risk groups respectively. The variable of age once again is demonstrated to have a significant negative correlation with BIS scores in the high risk group (beta = −0.283, *p* = 0.003), however this correlation was not significant in the low risk group. Conversely, when examining for an association between BDNF and BIS score, a significant positive association was identified in the low risk group (beta = 0.189, *p* = 0.014), which was absent from the high risk group (beta = −0.086, *p* = 0.361). None of the other variables examined, including BMI and sex had any significant association with BIS scores. The correlation between BDNF and BIS in each risk group is demonstrated visually in Figure 2.

## 4. Discussion

Our study aimed to determine whether serum levels of BDNF were associated with impulsivity score in an effort toward finding a measurable objective biomarker of impulsivity. Our findings suggest that BDNF is significantly associated with self-reported impulsivity scores on the BIS, though this was only true in our suicide low risk group. Our high risk group, as defined by patients admitted to hospital for suicide attempts, did not demonstrate this same correlation.

Many authors have proposed a direct association between suicide and BDNF levels, though a recent meta-analysis by Eisen and colleagues concludes that there no significant association between serum BDNF and attempted suicide [21]. Our study goal was to investigate BDNF as a marker of impulsivity that may indirectly be associated with suicidal behaviour.

Despite differences in suicidality between the two study groups, there was no difference in the BIS scores. Similar findings have been reported by Brezo et al., where the BIS was unable to discern between a population with suicidal ideation without any attempts from a population with suicide attempts [9]. Self-reported scales like the BIS carry inherent limitations and are highly subjective, and as such, have shown to be an inconsistent tool for stratifying risk and reliably identifying trait impulsivity. Our finding of an association between serum BDNF and self-reported impulsivity provides a potential for the use of BDNF as an objective tool to identify impulsivity, circumventing limitations of self-report scales.

BDNF has long been proposed to be neuroprotective, and many have proposed that low BDNF is associated with psychopathology [11]. In this study, we have demonstrated that high levels of serum BDNF are significantly correlated with an increase in self-reported impulsive behaviours. We propose that increased BDNF activity leads aberrantly high neurobiological activation, which leads to sudden and rapid behaviours without careful forethought. However in our study impulsivity was based on a score of a questionnaire and no actual impulsive behaviours were identified and thus the association between impulsivity as a trait measured on a continuous scale is likely different than impulsive behaviours such as suicidal behaviour which can be an impulsive act in some cases. Another limitation to this study is the multiple variables which have been shown to influence BDNF level, of which only sex, BMI and age controlled for.

The finding of a positive association between BDNF and impulsivity score was isolated to the control group, and was not present in the study group with recent suicide attempts. This finding may be explained by the subtlety of this association being lost on a population in a psychiatric hospital actively undergoing various treatments in response to suicide attempts. Such treatment measures may confound the self-reported impulsivity scores, and possibly even serum BDNF levels. Further research will be required to validate this hypothesis. Patients with recent serious suicide attempts have generally already presented to the attention of medical personnel, thus the real utility is identifying individuals who have high impulsiveness as a preventative measure before consequences of impulsive behaviour have a chance to contribute to major life stressors or suicide attempts.

Future applications of these findings include using the BDNF serum level as a clinical tool as part of a more comprehensive strategy to identify at risk individuals. Considering the modest association, serum BDNF is unlikely to replace clinical judgement but may ultimately enhance psychiatric risk evaluation. It would be useful to further explore whether serum BDNF levels are representative of chronic impulsivity, or if the levels are responsive to acute impulsivity such as in times of mental health crises. Developing a deeper understanding into the association between BDNF and impulsivity will guide how to most effectively capitalize on this as a biomarker.

## Figures and Tables

**Table 1:** Characteristics of study population.

Variable	Study Sample (*n* = 293)
Age (mean, standard deviation)	45.69 (15.52)
Sex (*n*, % female)	143 (48.80%)
Body Mass Index (mean, standard deviation)	34.42 (17.38)
Employment Status (*n*, % employed)	175 (60.34%)
Marital Status (*n*, % married)	95 (32.42%)
Children (*n*, % with children)	177 (60.41%)
Serum BDNF (mean, standard deviation) (pg/mL)	24,255.62 (7320.78)
Barratt Impulsiveness Scale score (mean, standard deviation)	65.72 (8.00)
Diagnoses	
Diagnosis of Mood Disorder (*n*, %)	101 (34.47%)
Diagnosis of Psychotic Disorder (*n*, %)	20 (6.83%)
Diagnosis of Substance Use Disorder (*n*, %)	69 (23.55%)
Diagnosis of Anxiety (or Anxiety Related) Disorder (*n*, %)	123 (41.98%)
Medications	
Treatment with Antidepressant (*n*, %)	122 (41.63%)
Treatment with Benzodiazepines (*n*, %)	132 (45.05%)
Treatment with Antipsychotic (*n*, %)	89 (30.38%)
Treatment with Mood Stabilizer (*n*, %)	65 (22.18%)
Ethnicity	
European (*n*, %)	240 (81.91%)
African (*n*, %)	25 (8.53%)
Asian (*n*, %)	9 (3.75%)
Native North or South American (*n*, %)	3 (1.02%)
Latin American (*n*, %)	2 (0.68%)
Middle Eastern (*n*, %)	5 (1.71%)
Other (*n*, %)	7 (2.39%)

**Table 2:** Characteristics of study populations by risk category.

Variable	High Risk (*n* = 117)	Low Risk (*n* = 176)	*p*
Age (mean, standard deviation)	44.96 (14.88)	46.17 (15.96)	0.749
Sex (*n*, % female)	53 (45.30%)	90 (50.13%)	0.326
Body Mass Index (mean, standard deviation)	32.92 (16.44)	34.62 (17.57)	0.629
Employment Status (*n*, % employed)	30 (25.86%)	85 (45.36%)	<0.001
Marital Status (*n*, % married)	32 (27.35%)	63 (35.79%)	0.130
Children (*n*, % with children)	71 (60.68%)	106 (60.22%)	0.938
Serum BDNF (mean, standard deviation)	23,771.56 (7088.10)	24,577.41 (7636.51)	0.947
Barratt Impulsiveness Scale score (mean, standard deviation)	65.76 (8.51)	65.69 (7.65)	0.731
Diagnoses			
Diagnosis of Mood Disorder (*n*, %)	81 (69.23%)	20 (11.36%)	<0.001
Diagnosis of Psychotic Disorder (*n*, %)	13 (11.11%)	7 (3.98%)	0.018
Diagnosis of Substance Use Disorder (*n*, %)	47 (40.17%)	22 (12.50%)	<0.001
Diagnosis of Anxiety (or Anxiety Related) Disorder (*n*, %)	89 (76.07%)	34 (19.32%)	<0.001
Medication			
Treatment with Antidepressant (*n*, %)	87 (74.36%)	35 (18.89%)	<0.001
Treatment with Benzodiazepines (*n*, %)	95 (81.20%)	37 (21.02%)	<0.001
Treatment with Antipsychotic (*n*, %)	63 (53.85%)	26 (14.77%)	<0.001
Treatment with Mood Stabilizer (*n*, %)	46 (39.32%)	19 (10.89%)	<0.001
Ethnicity			
European (*n*, %)	109 (93.16%)	131 (74.44%)	<0.001
African (*n*, %)	2 (1.71%)	23 (13.07%)	<0.001
Asian (*n*, %)	2 (1.71%)	9 (5.11%)	0.133
Native North or South American (*n*, %)	2 (1.71%)	1 (0.57%)	0.342
Latin American (*n*, %)	0 (0.00%)	2 (1.14%)	0.820
Middle Eastern (*n*, %)	0 (0.00%)	5 (2.84%)	0.105
Other (*n*, %)	2 (1.71%)	5 (2.84%)	0.534

**Table 3:** Multivariable linear regression with Barratt Impulsivity Scale score (*n* = 293).

Variable	Standardized Coefficient	95% CI		*p*-Value
		Lower	Upper	
Age	−0.168	−0.146	−0.027	0.004 *
Sex	0.069	−0.753	2.958	0.243
BMI	−0.027	−0.139	0.086	0.640
BDNF	0.090	−0.000028	0.00221	0.127
Risk Group	0.002	−1.849	1.913	0.973

BMI: Body Mass Index, BDNF: Brain-derived neurotrophic factor, CI: confidence interval. * Significant at *p* < 0.05.

**Table 4:** Multivariable Linear Regression for Low Risk Group (*n* = 176).

Variable	Standardized Coefficient	95% CI		*p*-Value
		Lower	Upper	
Age	−0.120	−0.129	0.014	0.113
Sex	0.050	−1.545	3.084	0.512
BMI	0.005	−0.129	0.138	0.947
BDNF	0.189	0.000038	0.000339	0.014 *

BMI: Body Mass Index, BDNF: Brain-derived neurotrophic factor, CI: confidence interval. * Significant at *p* < 0.05.

**Table 5:** Multivariable Linear Regression for High Risk Group (*n* = 117).

Variable	Standardized Coefficient	95% CI		*p*-Value
		Lower	Upper	
Age	−0.283	−0.269	−0.054	0.003 *
Sex	0.072	−1.887	4.354	0.435
BMI	−0.067	−0.276	0.128	0.471
BDNF	−0.086	−0.000328	0.000120	0.361

BMI: Body Mass Index, BDNF: Brain-derived neurotrophic factor, CI: confidence interval. * Significant at *p* < 0.05.
